# Implications of COVID-19 Outbreak on Immune Therapies in Multiple Sclerosis Patients—Lessons Learned From SARS and MERS

**DOI:** 10.3389/fimmu.2020.01059

**Published:** 2020-05-12

**Authors:** Nora Möhn, Refik Pul, Christoph Kleinschnitz, Harald Prüss, Torsten Witte, Martin Stangel, Thomas Skripuletz

**Affiliations:** ^1^Department of Neurology, Hanover Medical School, Hanover, Germany; ^2^Department of Neurology, University Hospital Essen, Essen, Germany; ^3^Department of Neurology, Charité Universitätsmedizin Berlin, Berlin, Germany; ^4^German Center for Neurodegenerative Diseases (DZNE), Berlin, Germany; ^5^Department of Rheumatology and Immunology, Hannover Medical School, Hanover, Germany

**Keywords:** SARS, MERS, COVID-19, multiple sclerosis, immunosuppressive therapy, DMTs

## Abstract

Severe acute respiratory syndrome coronavirus-2 (SARS-CoV-2) pandemic keeps the world in suspense. In addition to the fundamental challenges for the health care system, the individual departments must decide how to deal with patients at risk. Neurologists are confronted with the question, how they should advise their patients regarding immunosuppressive treatment. In particular, the large number of different disease-modifying therapies (DMTs) in the treatment of neuroimmunological diseases such as multiple sclerosis poses a challenge. To a limited extent, it might be useful to transfer knowledge from previous SARS- and Middle East respiratory syndrome (MERS) coronavirus outbreaks in 2002/2003 and 2012 to the current situation. Overall, immunosuppressive therapy does neither seem to have a major impact on infection with SARS- and MERS-CoV nor does it seem to lead to a severe disease course in many cases. Considering the immunological responses against infections with novel coronaviruses in humans, interferons, glatiramer acetate, and teriflunomide appear to be safe. As lymphopenia seems to be associated with a more severe disease course, all DMTs causing lymphopenia, such as cladribine, alemtuzumab, and dimethyl fumarate, need to be reviewed more thoroughly. As they are, in general, associated with a higher risk of infection, depleting anti-CD20 antibodies may be problematic drugs. However, it has to be differentiated between the depletion phase and the phase of immune reconstitution. In summary, previous coronavirus outbreaks have not shown an increased risk for immunocompromised patients. Patients with severe neuroimmunological diseases should be kept from hasty discontinuation of immunotherapy.

## Introduction

The world, and especially our healthcare system, is currently confronted with one of the greatest challenges of modern times. As of April 17, 2020, 2,165,500 people have been infected with the severe acute respiratory syndrome coronavirus 2 (SARS-CoV-2). The resulting disease, designated as coronavirus disease 2019 (COVID-19), has cost 53,164 people their lives so far (Johns Hopkins database, accessed: 17.04.2020, https://coronavirus.jhu.edu/map.html). Advanced age and pulmonary comorbidities are known risk factors for a severe clinical course with possible fatal outcome. However, the role of immunosuppressive medications as a potential risk factor especially in neuroimmunological disorders such as multiple sclerosis (MS), chronic inflammatory demyelinating polyneuropathy (CIDP), autoimmune encephalitis, myasthenia gravis, Neuro-Sjögren, cerebral vasculitis, or neuromyelitis optica spectrum disorder (NMOSD) is still not clear.

## Disease Modifying Therapies

Aside from treatment of exacerbations and symptomatic therapies, slowing disease progression with disease-modifying therapies (DMTs) is particularly relevant. MS is an inflammatory disease of the central nervous system (CNS) causing damage to the myelin sheath and leading to axonal destruction (1). DMTs are immunosuppressive agents which inhibit the exaggerated immune response (2). Currently, 11 drugs (namely intramuscular interferon (IFN) beta-1a; subcutaneous IFN beta-1a; subcutaneous IFN beta-1b; subcutaneous glatiramer acetate; oral dimethyl fumarate; oral teriflunomide; oral fingolimod; oral cladribine; intravenous natalizumab; intravenous alemtuzumab; and intravenous ocrelizumab) are approved for the treatment of relapsing-remitting MS (RRMS) in the European Union. The monoclonal antibody ocrelizumab is the only approved therapy for primary-progressive MS (PPMS). Siponimod was introduced to the market for treatment of secondary-progressive MS (SPMS) in the EU. Recently, a nationwide Swedish cohort study has shown that MS patients are at a generally increased risk of infection (3). Results of this large observational study suggested that rituximab was the only DMT with a significantly increased rate of infections compared with interferon beta and glatiramer acetate in the most adjusted model. This included especially severe bacterial infections. Fingolimod and natalizumab showed a trend toward an increased rate of infection compared with interferon beta and glatiramer acetate, but no significance was found (3). Although 6,421 patients have been included in this study, its findings must be considered with caution, as it is a single register-based cohort study.

## Findings During SARS-CoV and MERS outbreak

When aiming for recommendations on (dis)continuation or change of DMT in immunosuppressed patients in times of the COVID-19 pandemic, we have to focus on what is known about immunological responses against (SARS-) coronavirus infections in humans (4). Moreover, we should be guided by the findings of SARS- and Middle East respiratory syndrome (MERS) coronavirus outbreaks in 2002/2003 and 2012, respectively, regarding immunosuppression as a relevant risk factor. Partial conclusions for the current situation might then possibly be drawn.

### Immunological Processes During SARS-CoV Infection

During the SARS-CoV outbreak 2002–2003 which resulted in 916 deaths among more than 8,098 infected patients in 29 countries, those infected developed a mild to fatal pulmonary disease (fatality rate of more than 10%) (5). In patients with severe disease and worse outcomes, a more protracted course with lymphopenia, neutrophilia, and prolonged cytokine production was observed. Additionally, those patients had a slightly higher leukocyte count than patients who did not develop severe pulmonary disease (5–7). A limited and delayed virus elimination due to suboptimal T and B cell response was assumed to be responsible for severe disease courses. However, no correlation between disease activity and viral load was observed (5–7). Of note, more than 95% of SARS-CoV infected patients presented with specific IgG antibodies 25 days after the onset of viral infection (8). The protective effect of humoral immunity is mainly based on neutralizing antibodies which impede the virus to enter the host cells (4). In case of SARS-CoV, neutralizing antibodies are directed against the spike (S) glycoprotein which mediates membrane fusion between virus and host cell (9). In patients with severe disease suboptimal neutralizing antibody responses could be detected (10). Cytotoxic T lymphocytes (CTL) are essential for clearing respiratory viruses and provide a robust protective cellular immunity (11). It is known that infection with SARS-CoV induces long-lasting T cell response in surviving humans (12). Studies have shown that epitope-specific CD8^+^ T cells are crucial for protection upon SARS-CoV reinfection as specific antibody response might eventually disappear (13, 14).

### Immunological Processes During MERS-CoV Infection

MERS-CoV was initially discovered in Saudi Arabia in 2012 (15). The World Health Organization (WHO) has confirmed 2,279 cases of human infections with MERS-CoV in 27 countries since 2012, whereby up to February 2019, 806 (35%) infected patients have died (16). The mechanisms of the immune response triggered by MERS-CoV infection and immune evasion strategies have not yet been fully elucidated. It has been shown that MERS-CoV induces immunosuppression to escape the host's immune surveillance, partly by promoting T-cell apoptosis. Studies indicate that MERS-CoV has also evolved strategies to inhibit innate immunity and IFN production pathways. The complex mechanisms include for example the fact that negative regulators of transcription factors inducing INF-α and INF-β are upregulated during MERS-CoV infection (17). In 2013 researchers demonstrated that *in vitro* treatment with INF-α could have some beneficial effects on MERS-CoV infected cells (18). Others showed a potent inhibitory effect of INF-β on MERS-CoV *in vitro* (19). Regarding the adaptive immune system, little is known about what constitutes a protective immune response in MERS patients who recovered (20). Similar to SARS-CoV, MERS-CoV seems to elicit attenuated innate immune responses with delayed pro-inflammatory cytokine induction, namely IFN- γ and IL-12, in cell culture and *in vivo* (14, 21, 22).

### Immunosuppression and Coronavirus Infection

When analyzing potential risk factors of infection and severe disease course during the SARS- and MERS-CoV outbreaks, risk factors for both infections included advanced age, male sex, and the presence of co-morbidities (for example obesity, diabetes mellitus, heart disease, arterial hypertension, lung disease) (20, 23). Detailed investigations about patients with an immunocompromised state and especially immunosuppressive treatment are lacking, though. In some studies, individual patients with reduced immune status were mentioned. A case series about 12 critically ill MERS-CoV patients reported one patient suffering from malignant melanoma and one patient who had received kidney and liver transplant (24). Another study described 47 MERS-CoV patients of which 45 (96%) had underlying comorbid medical disorders. One patient of those 45 was on long-term immunosuppressive treatment with steroids (25). Al-Abdallat and colleagues found no evidence of underlying immunodeficiency or immunosuppressant medications and therapies among any of their subjects (*n* = 9) during a hospital-associated MERS-CoV outbreak (26). Overall, immunosuppressive therapy does neither seem to have a major impact on infection with SARS- and MERS-CoV nor does it seem to lead to a severe disease course in many cases (23). However, it has to be kept in mind that reported case numbers are very small.

Available data on the current COVID-19 pandemic show similar results. A retrospective cohort study about risk factors for death in adults in Wuhan could identify advanced age, d-dimer levels >1 μg/ml, and a high Sequential Organ Failure Assessment (SOFA) Score on admission (27). In Bergamo, Italy, clinicians found out that children under the age of 12 did not develop severe pneumonia, regardless of their immune status and concluded that immunosuppressed patients are not at increased risk of severe pulmonary disease compared to the general population (23).

## Discussion

So, what conclusions can we draw for our immunosuppressed MS—and potentially further neuroimmunological—patients? Of course, most of the high-efficient DMTs had not been approved during SARS- and MERS-CoV outbreak. Consequently, we have no data regarding the risk for those patients and can only speculate about possible mechanisms. Overall, there is little data about specific immunosuppressant/immunomodulatory drugs and their potential impact on susceptibility to infection with novel coronaviruses. The general observations on past and present coronavirus outbreaks suggest that advanced age, male sex, obesity, high blood pressure, and other comorbidities are more relevant than an immunosuppressed status, regarding the risk of infection and of severe disease course.

Considering the immunological responses against infections with novel coronaviruses in humans, interferons, and glatiramer acetate should not pose an increased risk of infection. Interferons may even be protective as beneficial effects were found in *in vitro* experiments (18, 19, 28, 29). Since glatiramer acetate is known to induce T helper cells and regulatory T cells (30), it might not to be assumed that there is an increased risk of serious infections under this medication. Studies on teriflunomide provide evidence that it does not have a negative impact on protective immunity (31). Since elevated interleukin- (IL-6) levels have been detected in severe diseased COVID-19 patients (32) and teriflunomide is thought to decrease the release of proinflammatory cytokines like IL-6, IL-8, and monocyte-derived chemotactic protein-1 (MCP-1) from monocytes, it could even have a positive effect in case of SARS-CoV-2 infection. Furthermore, studies on Theiler's murine encephalomyelitis virus (TMEV) infected mice have shown that teriflunomide treatment leads to increased viral clearance and reduced serum TEMV antibody concentrations (33–35). Natalizumab prevents the transmigration of T lymphocytes across the blood-brain barrier by blocking the alpha-4 subunit of integrin molecules. It might be assumed that natalizumab treatment will not have a markedly negative effect on SARS-CoV-2 infected patients. As in the SARS-CoV epidemic of 2002/2003, COVID-19 patients with severe disease exhibit significant lymphopenia, whereby especially T cell count is reduced (32). We might conclude that DMTs which induce pronounced lymphopenia have unfavorable implications on COVID-19 disease course. These include cladribine, alemtuzumab, and to a lesser extent dimethyl fumarate. Anti-CD20-antibodies ocrelizumab and rituximab mainly deplete B lymphocytes. However, CD20 is also expressed at a low level on a subset of T cells (36). CD20^+^ T cells represent a highly activated subpopulation with enhanced cytokine production even during resting conditions and might thus play a crucial role in pro-inflammatory processes (37). Furthermore, compared with other DMTs, anti-CD20-antibodies entail a higher risk of infections, especially with bacteria (3). In the context of SARS-CoV-2 infection, bacterial co-infections may be associated with a more severe disease course. Additionally, CD20-antibodies could impede the production of neutralizing antibodies which might lead to a protracted course of disease and a worse outcome. However, in depleting therapies it has to be differentiated between the depletion phase and the phase of immune reconstitution. The latter could in turn lead to increased tissue damage in infected patients due to a rather excessive immune response. On the other hand, such mechanisms may benefit an exaggerated immune response against the virus, and stopping or changing those DMTs may hinder viral clearance. Treatment with fingolimod is associated with an increased risk for bradyarrhythmia and atrioventricular blocks during treatment initiation, elevated liver function tests, and an increased risk of infections, including herpes simplex, cryptococcal, and varicella zoster viral infections [Gilenya (fingolimod) prescribing information, Novartis 2016]. Sphingosine-1-phosphate (S1P) receptor modulators are generally associated with an elevated risk of respiratory tract infections (38). Together with their cardiac side effects, this might have a negative impact on SARS-CoV-2 infection rates and COVID-19 disease course.

As a final consideration, it should be noted that coronaviruses seem to implicate the inflammatory host response as an important contributor to the disease process. Dysregulated (innate) immune responses appear to be crucial drivers of tissue damage after the initial infection (23). Thus, immunomodulating therapy might not only be seen as a risk factor, but could help to attenuate the damage caused by viral induced excessive immune response.

All those speculations need to be proven in clinical studies which will investigate the impact of COVID-19 in patients with MS and other neuroimmunological diseases on the clinical course and the influence of DMTs. Until then, the lack of major disease aggravation by DMTs according to the available experience and the even potentially beneficial effects of some DMTs against excessive viral-induced inflammation should detain patients with neuroimmunological diseases from hasty discontinuation of immunotherapy. Patients who are stable under current immunomodulatory therapy should continue their medication. The risk of disease activity with consecutive hospitalization currently appears more threatening than the risk of possible SARS-CoV2 infection in patients under DMTs. In patients with active neuroimmunological diseases such as MS, based on the limited data available, cell-depleting therapy currently should be considered with greater caution.

## Data Availability Statement

The original contributions presented in the study are included in the article/supplementary materials, further inquiries can be directed to the corresponding author.

## Author Contributions

TS and NM contributed to the conception and design of the study. NM wrote the first draft of the manuscript. TS, RP, CK, HP, TW, and MS revised the manuscript and contributed valuable intellectual content. All authors contributed to manuscript revision, read and approved the submitted version.

## Conflict of Interest

Outside the submitted work, the authors received honoraria for lectures, travel grants, or research grants. NM received honoraria for lectures from Merck and Novartis. RP and CK received honoraria for lectures and travel grants from Alexion, Bayer, Biogen, Celgene, MedDay, Merck, Mylan, Novartis, Roche, Sanofi Aventis, and TEVA. RP and CK received research support from Novartis and Merck. HP received honoraria for lectures from Euroimmun, Fresenius and Roche. TW received honoraria for lectures and travel grants from Abbvie, Amgen, Biogen, Boehringer Ingelheim, Celgene, Chugai, CSL Behring, Gilead, GSK, Janssen, Lilly, MSD, Mylan, Novartis, Pfizer, Roche, UCB, and Sanofi Aventis. MS received honoraria for lectures and travel grants from Alexion, Bayer, Biogen, Celgene, CSL Behring, Grifols, Janssen, MedDay, Merck, Novartis, Roche, Sanofi Aventis, Takeda, and TEVA. His institution received research support from Sanofi Aventis and Merck. TS received honoraria for lectures and travel grants from Alexion, Bayer, Biogen, Celgene, CSL Behring, Euroimmun, Merck, Novartis, Roche, Sanofi Aventis.

The authors declare that the research was conducted in the absence of any commercial or financial relationships that could be construed as a potential conflict of interest.
